# Physical mechanisms of human brain functions

**DOI:** 10.1002/qub2.70

**Published:** 2024-12-07

**Authors:** Zonghua Liu

**Affiliations:** ^1^ School of Physics and Electronic Science East China Normal University Shanghai China

**Keywords:** chimera states, eigenmode analysis, network synchronization, remote firing propagation, remote synchronization

## Abstract

In recent years, exploring the physical mechanisms of brain functions has been a hot topic in the fields of nonlinear dynamics and complex networks, and many important achievements have been made, mainly based on the characteristic features of time series of human brain. To speed up the further study of this problem, herein we make a brief review on these important achievements, which includes the aspects of explaining: (i) the mechanism of brain rhythms by network synchronization, (ii) the mechanism of unihemispheric sleep by chimera states, (iii) the fundamental difference between the structural and functional brain networks by remote synchronization, (iv) the mechanism of stronger detection ability of human brain to weak signals by remote firing propagation, and (v) the mechanism of dementia patterns by eigen‐microstate analysis. As a brief review, we will mainly focus on the aspects of basic ideas, research histories, and key results but ignore the tedious mathematical derivations. Moreover, some outlooks will be discussed for future studies.

## 1 INTRODUCTION

Roughly speaking, we have been trying to study the physical mechanisms of human brain functions throughout human history and many empirical evidences have been collected so far, including from both overseas and domestic. In general, these empirical evidences are occasionally found from time to time. For example, in overseas, it was occasionally revealed by the case of Henry Molaison (H.M.) that the hippocampus takes charge of memory and learning. Similarly, by the case of Phineas Gage, it was revealed that the frontal lobe takes charge of personality. And by the case of William Jenkins (W.J.), it was revealed that our two cerebral hemispheres are connected by the corpus callosum [1]. While in the domestic, it was noticed that our brain can be seriously influenced by unexpected emotional events, such as the phenomena of “hair turned into white overnight” and “people are in high spirits when involved in happy events”. In this way, our understanding to cognitive and memory functions is gradually but slowly increased. Although these achievements, we still have countless mysteries such as why someone is smarter than us or why we are not so happy as others.

However, when time comes to the modern times, our understanding to cognitive and memory functions is extremely speeded up, mainly due to the development of modern physical techniques such as the electroencephalogram (EEG), functional magnetic resonance imaging (fMRI), magnetoencephalogram, and blood oxygen level‐dependent. Based on these techniques, we now know that the human brain is very energy intensive. It only accounts for 2% of the total body mass, but consumes 20% of the total energy, where most of which is consumed by the ongoing neuronal signaling and the task‐related consumption is much less than the resting energy consumption (<5%) [2]. Moreover, it is now well known that the brain neural network is very complicated, with about 10^11^ neurons and 10^14^ links among these neurons [3, 4].

**FIGURE 1:**
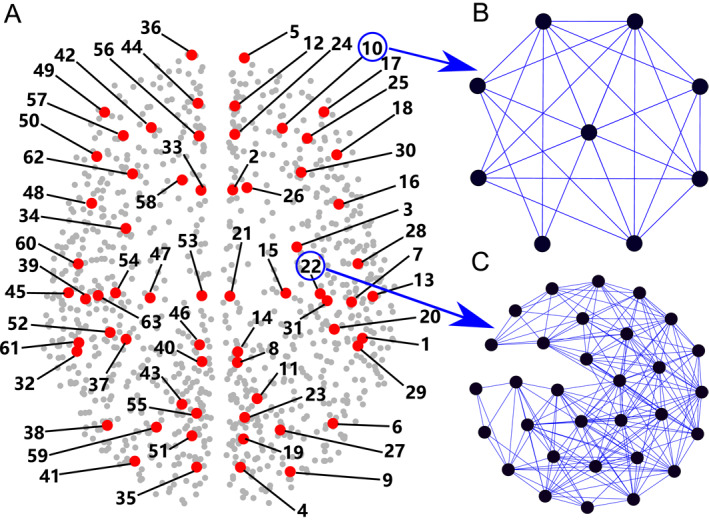
The global brain network and its local brain networks. (A) Represents the global brain network where the gray background points represent its nodes and the red points represent the central positions of the 63 local brain networks. (B) Represents the 10th local brain network. And (C) represents the 22th local brain networks. Reproduced from Ref. [10] with permission.

It is very hard to study the dynamics of this huge number of neurons. A convenient way is to simplify the brain neural network into a structural/anatomic brain network with much less size. For this purpose, many approaches have been proposed and many structural brain networks have been obtained, such as the networks with 998 nodes [5, 6], 234 nodes [7], and 76 nodes [8], respectively. By these networks, it is revealed that the structural brain network has the features of both small‐world and community [9]. Take the brain network of 998 nodes as an example. According to the data of Refs. [5, 6], the cerebral cortex is divided into different regions of interest (ROIs) and each ROI is considered as a node. The connection between any two nodes is measured noninvasively by using diffusion spectrum imaging, resulting in totally 17,865 links. Further, this network can be divided into 63 different local brain networks. In each of them, the connections among the nodes in the same local brain network are kept while the connections to other local brain networks are removed. The gray background points of Figure 1A show all the nodes locations on the global brain network, and the red points of Figure 1A are the central positions of the 63 local brain networks, which are ordered in sequence by a number *i* with *i* = 1,…, 63 [10]. For details, the topologies of the 10th and 22th local brain networks are shown in Figure 1B,C, respectively. It is easy to see that they have different topologies, indicating the significant difference among different local brain networks.

**FIGURE 2:**
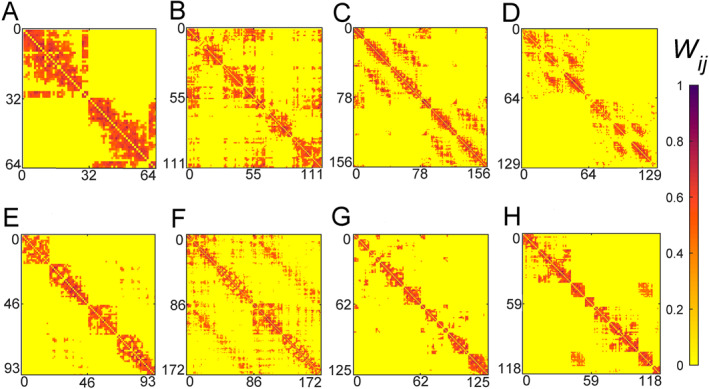
The connection matrixes of the eight cognitive subnetworks extracted from the whole brain network where the color bar represents the weights *W*_*ij*_. (A–H) Represent the weight matrixes of the eight cognitive subnetworks of Aud, V, MS, VT, Att, mDm, CO, and FP systems, respectively. Reproduced from Ref. [17] with permission. Att, attention system; Aud, auditory; CO, cingulo‐opercular; FP, frontoparietal; mDm, medial default mode; V, visual; VT, ventral temporal association.

In general, a specific brain function will be performed by one or a few cognitive subnetworks but not the whole brain network. For example, when human brain receives the signals of light, sound, taste, and smell, their corresponding cognitive subnetworks of visual, auditory, gustatory cortex, and olfactory cortex will be automatically activated, respectively. This topology of brain network is benefit for the segregation and integration of cognitive processes [11, 12]. Various brain functions can be efficiently performed under the condition of optimal balance between functional integration and segregation. Correspondingly, we can divide the brain network into different cognitive subnetworks. To figure out the network topologies of cognitive subnetworks, it is noticed that the activity of certain regions of brain is closely correlated with the cognitive task, whereas others decrease activity [13, 14, 15, 16]. Surprisingly, it is further noticed that there is correlation among the regions similarly modulated by tasks or stimuli, even in the absence of tasks or stimuli. In this way, each cognitive subnetwork can be defined by the regions that coactivate in the similar cognitive functions [8]. For example, we may classify the brain network into eight cognitive subnetworks, named as auditory (Aud), visual (V), motor and somatosensory (MS), ventral temporal association (VT), attention system (Att), medial default mode (mDm), cingulo‐opercular (CO), and frontoparietal (FP) systems, respectively [8, 17]. To extract a specific cognitive subnetwork from the whole brain network, we may consider only the connections within the cognitive subnetwork but remove all those connections to other cognitive subnetworks. Figure 2A–H show the weighted connection matrixes *W*_*ij*_ of the extracted eight cognitive subnetworks, respectively.

Alternatively, we may have another brain network, that is, the functional brain network, in contrast to the structural brain network. The functional brain network represents the functional relationship between different brain regions and is measured by the Pearson correlation coefficients between the EEG time series of nodes [18]. Thus, the functional brain network reflects the dynamic interaction of functionally specialized but widely distributed cortical regions, that is, it is not static but time dependent [19, 20]. In details, we may obtain the functional brain network as follows Ref. [21]. For each node *x* of the structural brain network, we measure its time series as *V*(*x, t*). A functional connection between two nodes can be defined when their temporal correlation exceeds a threshold *r*_*c*_, regardless of their structural connectivity. Then, we calculate their Pearson correlation coefficient by

(1) $$r\left(x_{1},x_{2}\right)=\frac{\langle V\left(x_{1},t\right)V\left(x_{2},t\right)\rangle -\langle V\left(x_{1},t\right)\rangle \langle V\left(x_{2},t\right)\rangle }{\sigma (V\left(x_{1}\right))\sigma (V\left(x_{2}\right))}$$

where *σ* ^2^(*V*(*x*)) = 〈*V*(*x*,*t*)^2^〉 − 〈*V*(*x*,*t*)〉^2^, and 〈•〉 represents temporal averages. A key point is the choose of the threshold *r*_*c*_, where networks of different sparsity or connection density will be obtained by different thresholds.

Comparing the functional brain network from Equation (1) with the structural brain network, it is found that they are significantly different with each other, thus their relationship is currently an active research area [22]. One of their characteristic differences is that the functional brain network has much more long connections than the structural brain network. To explain the mechanisms of these differences, many new research directions have been gradually developed, including the network synchronization, remote synchronization (RS), chimera states, firing propagation, and eigenmode analysis, aiming to explain the mechanisms of brain rhythms, long connections of functional brain network, unihemispheric sleep, stronger detection ability of weak signals, and patterns of dementia, respectively. These results have extremely increased our understanding to the mechanisms of human brain functions. To promote the further study of this field, herein we make a brief review of these progresses on the physical mechanisms of brain functions. To avoid the tedious mathematical derivations, we will mainly focus on their basic ideas, research histories, and key achievements. Moreover, we will discuss some outlooks for future studies.

## 2 BRAIN RHYTHMS AND NETWORK SYNCHRONIZATION

Biorhythm is a common phenomenon in our daily life such as the 24 h circadian rhythm and woman’s menstrual cycle. However, it is revealed by EEG data that human brain also has other featured rhythms, named as alpha, beta, gamma, theta, and delta rhythms, respectively, where various oscillating networks of cortical neurons will be formed [23]. For examples, it is revealed that neural correlates of visual awareness may lead to synchronous neural firing at gamma frequencies [24]. While neuronal networks in the mammalian forebrain may span five orders of frequency bands, that is, from 0.05 to 500 Hz [23]. Moreover, these specific rhythms are found to be closely related to particularly cognitive processes: global synchronization at the gamma frequency is related with consciousness, alpha and gamma rhythms are related with attentional suppression and focusing, and theta and gamma rhythms are related with memory encoding and retrieval etc. [25].

Especially, even in sleep, it is found that we have a repeated 90‐min ultradian cycle of rapid‐eye‐movement (REM) sleep and non‐rapid‐eye movement (NREM) [26, 27]. In each ultradian cycle, we generally have five stages: the first stage with the alpha rhythm (8−13 Hz), second stage with the theta rhythm (4−7 Hz), third and fourth stages with the delta rhythm (0−4 Hz), and fifth stage with the beta rhythm (13−30 Hz). The fifth stage is in fact the REM sleep where we will have dreams. The third and fourth stages are also named as slow‐wave sleep where multiple subcortical targets can be recruited [28]. Very interesting, it was found that the oscillatory patterns in sleep are related to the experiences during the previous awake period [29, 30].

To understand the mechanisms of these brain rhythms, an assumption is that these rhythms are generated by network synchronization. Many evidences have been discovered to support this assumption. For example, it is found that each individual brain function is performed by only one or a few specific functional subnetworks [31, 32, 33]. Especially, epileptic seizure represents an abnormal synchronization spreading on the whole brain network [34]. Therefore, the mechanisms of brain rhythms can be revealed through the study of network synchronization. In this way, the network synchronization has gotten enough attention in the past decades and many different forms of synchronization have been revealed, such as the complete synchronization, phase synchronization, delay synchronization, generalized synchronization, partial synchronization, and explosive synchronization. [35, 36, 37]. Surprisingly, all these different forms of synchronization have been observed in the time series of human brain.

**FIGURE 3:**
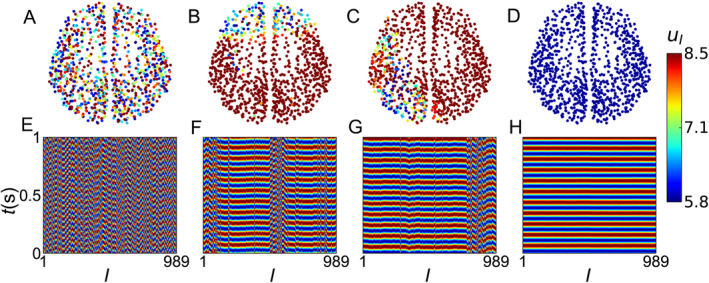
Various dynamical patterns for different pairs of *c* and *τ*. (A–D) Show four typical behaviors and (E–H) show their corresponding evolutions, where (A, E) represent the case of incoherent state, (B, F) represent the case of multiple chimera state, (C, G) represent the case of unihemispheric sleep, and (D, H) represent the case of synchronization. Adapted from Ref. [33].

In the field of synchronization, a key point is to figure out the critical coupling strength where a coherent motion of coupled oscillators will emerge once the coupling strength exceeds this critical coupling. To discuss the global synchronization of human brain network, Huo et al. considered a mean‐field population model [33] where the nodes and connections of network are from the data of Figure 1 and the nodes dynamics are chosen as the neural mass model [38, 39]. In details, the networked neural mass model can be represented as

$$\frac{{\text{d}}^{2}v_{I}^{p}}{\text{d}t^{2}}=Aaf\left(v_{I}^{e}-v_{I}^{i}\right)-2a\frac{\text{d}v_{I}^{p}}{\text{d}t}-a^{2}v_{I}^{p},$$

$$\frac{{\text{d}}^{2}v_{I}^{i}}{\text{d}t^{2}}=BbC_{4}f\left(C_{3}v_{I}^{p}\right)-2b\frac{\text{d}v_{I}^{i}}{\text{d}t}-b^{2}v_{I}^{i},$$

(2) $$\frac{{\text{d}}^{2}v_{I}^{e}}{\text{d}t^{2}}=Aa\left[C_{2}f\left(C_{1}v_{I}^{p}\right)+p_{I}+\frac{c}{\lambda_{I}}\sum_{J=1}^{N}M_{IJ}f\left(v_{J}^{e}\left(t-\tau \right)-v_{I}^{e}\right)\right]-2a\frac{\text{d}v_{I}^{e}}{\text{d}t}-a^{2}v_{I}^{e},$$

where *v* ^*p*^ *, v* ^*i*^ and *v* ^*e*^ represent the post‐synaptic membrane potentials of the node‐*I* for the groups of pyramidal neurons, inhibitory and excitatory interneurons, respectively. *f*(*v*) is the sigmoid function. *M*_*IJ*_ is the weighted coupling matrix from the data of Figure 1. *c* is the overall coupling strength and *τ* is the time‐delay. A detailed interpretation for all the other parameters can be found in Ref. [33]. It is found that various dynamics patterns can be observed for different parameter settings *τ* − *c*. Figure 3A–D show four typical behaviors and (E–H) show their corresponding evolutions, where (A) and (E) represent the case of incoherent state, (B) and (F) represent the case of multiple chimera state, (C) and (G) represent the case of unihemispheric sleep with the nodes on the right cerebral hemisphere being synchronized while the others on the left cerebral hemisphere being disordered, and (D) and (H) represent the case of synchronization, which corresponds to the abnormal synchronization of epileptic seizure.

In addition to the stabilized patterns such as those in Figure 3, many cognitive brain functions rely on dynamic patterns and can frequently switch between different firing patterns so that to implement different tasks. This kind of switching of brain dynamics even happens during sleep cycles or memory recall and is known as different rhythms. It was found that the synchronization of *α* and *β* rhythms between right inferior frontal and primary sensory neocortex has been associated with attentional control [40]. And higher frequency *γ* rhythms in local synchronization have been observed during visual responses [41]. Especially, the rhythm of resting‐state networks will be frequently interrupted by the sleep‐wake switch producing stable sleep and wakefulness [42]. More examples can be found in pathologies such as epilepsy, autism spectrum disorders, schizophrenia or Alzheimer’s disease (AD) where there is frequent switching between diseases and normal states [43, 44]. To implement this switching, Huo et al. presented a framework of reaction‐diffusion model [45]. In the reaction step, each activated node will have a probability *p* to become inactivated and 1 – *p* to remain in activated state. That is, we have

(3) $$x_{i}\left(t+1\right)=\left\{\begin{array}{ccc} 0, & \text{with} & p\\ 1, & \text{with} & 1-p \end{array}\right.$$

for activated *x*_*i*_(*t*) = 1. In the diffusion step, an inactivated node will become activated when its total input coupling strength is greater than a threshold *w*_*c*_. The process can be represented as

(4) $$x_{i}\left(t+1\right)=\Theta \left(\sum_{j=1}^{N}W_{ij}x_{j}\left(t\right)-w_{c}\right)$$

for inactivated *x*_*i*_(*t*) = 0, where Θ denotes the Heaviside function.

**FIGURE 4:**
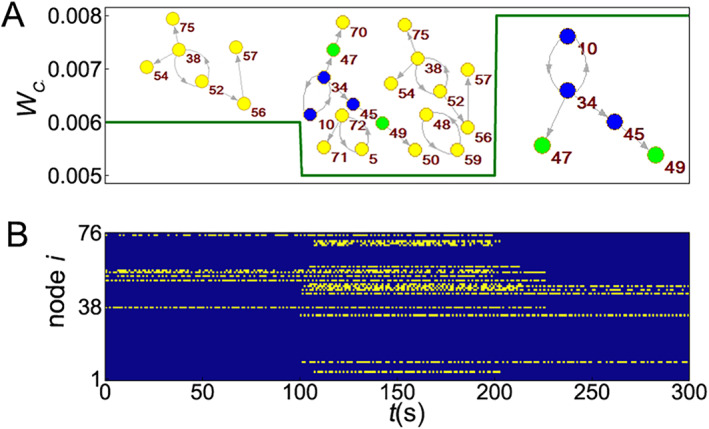
Switching between different rhythms by a time‐dependent threshold. (A) Time‐dependent threshold *w*_*c*_ where the threshold *w*_*c*_ is chosen as 0.006 for *t <* 100, 0.005 for 100 < *t <* 200, and 0.008 for 200 < *t <* 300, respectively. (B) The evolutionary process of the dynamics patterns where the threshold *w*_*c*_ is changed according to (A). Adapted from Ref. [45].

Huo et al. considered a brain subnetwork with *N* = 18 [45] and found that the switching between different rhythms can be implemented, provided that the threshold *w*_*c*_ is time‐dependent. Figure 4 shows the result where the variation of *w*_*c*_ is given in (A) and the corresponding dynamics patterns are given in (B). We see from Figure 4B that the pattern changes with time, indicating that the switching between different rhythms has been implemented by a time‐dependent activating threshold.

An important finding of Figure 3 is that the system dynamics before synchronization is not always disordered but may have rich patterns, depending on the setting of *τ* − *c*. Recently, based on eigenvector‐based analysis, Fan et al. showed that some of these patterns will form cluster synchronization and the emergence and transition of the cluster synchronization states can be theoretically predicted by the eigenvector‐based analysis [46]. They considered empirical neural networks and focus on the transition regime with weaker couplings. Their interesting finding is that there are critical couplings for the patterns of cluster synchronization. The framework of eigenvector‐based analysis mainly contains four steps as follows [46]:

1. In the first step, we consider the dynamics of a general network
(5) $$\frac{dx_{i}}{dt}=F\left(x_{i}\right)+\varepsilon \sum_{j=1}^{N}W_{ij}H\left(x_{j}\right)$$
  where *F*(*x*) represents the dynamics of oscillators, *ε* is the coupling strength, *H*(*x*) is the coupling function, and *W* = {*W*_*ij*_} represents the network connection matrix with *W*_*ij*_ denoting the connection weight between two connected nodes *i* and *j*. Specifically, *W*_*ii*_ is defined as W_*ii*_ = −∑_*j*(*j*≠*i*)_ *W*_*ij*_, thus *W* represents the Laplacian matrix. According to the approach of master stability function [47], we obtain the variational equations
(6) $$\frac{\delta dx_{i}}{dt}=DF\left(s\right)\delta x_{i}+\varepsilon \sum_{j=1}^{N}W_{ij}DH\left(s\right)\delta x_{j}$$
  with *s* being the global synchronization manifold. Equation (6) can be further rewritten by the eigenvectors of *W* as
(7) $$\frac{\delta dy_{i}}{dt}=\left[DF\left(s\right)+\varepsilon \lambda_{i}DH\left(s\right)\right]\delta y_{i}$$
  where 0 = *λ*_1_ > *λ*_2_ ≥ … ≥ *λ*_*N*_ represent the eigenvalues of *W*.
2. In the second step, we let σ≡−ελ, thus Equation (7) becomes
(8) $$\frac{\delta dy_{i}}{dt}=\left[DF\left(s\right)-\sigma_{i}DH\left(s\right)\right]\delta y_{i}$$
  which is the master equation of the perturbation dynamics [47]. By solving Equation (8), we can obtain the largest conditional Lyapunov exponent Λ, which will change with *σ*. The system will be stable for Λ < 0 and unstable for Λ > 0. Thus, there is a critical point of *σ*_*c*_ in which Λ becomes negative when *σ* > *σ*_*c*_. Equivalently, there is a critical coupling *ε*_*c*_ where all the oscillators will be synchronized when *ε* > *ε*_*c*_ = −*σ*_*c*_/*λ*_2_.
3. In the third step, we focus on the desynchronization state with *ε* < *ε*_*c*_, in contrast to the synchronization state in previous studies. Especially, we here only consider the situation when the first mode in the transverse space is unstable. When *ε* is gradually decreased from *ε*_*c*_, the mode *δ* *y*_2_ will be firstly emerged from the synchronized state. Let *δ* *y*_2_ be the mode of *λ*_2_ and *v*_2_ be the eigenvector associated with *λ*_2_. We have
(9) $$\delta x_{i}\left(t\right)=c\left(t\right)+v_{2,i}\delta y_{2}\left(t\right)$$
  with *c*(*t*) being a variable from the mode *δ* *y*_1_.
4. Finally, in the fourth step, we check the values of *v*_2*,i*_ for all the different *i.* If *v*_2*,i*_ = *v*_2*,j*_, the oscillators *i* and *j* will have no difference during the evolutionary process and thus be completely synchronized. In this way, the group of oscillators with the same eigenvector element will form a synchronized cluster.

This is an efficient way to figure out all the synchronization clusters when the mode of *λ*_2_ becomes unstable. Discussions on more unstable modes can be found in Ref. [46]. However, this framework will fail when the oscillators are nonidentical. Moreover, a key feature of brain network is its community topology where the coupling strength for the connections within the same community is stronger but weaker for those between communities. That is, their coupling strengths are heterogeneously distributed. Therefore, we need to study the synchronization of cognitive subnetworks [19], in contrast to the synchronization of global brain network.

**FIGURE 5:**
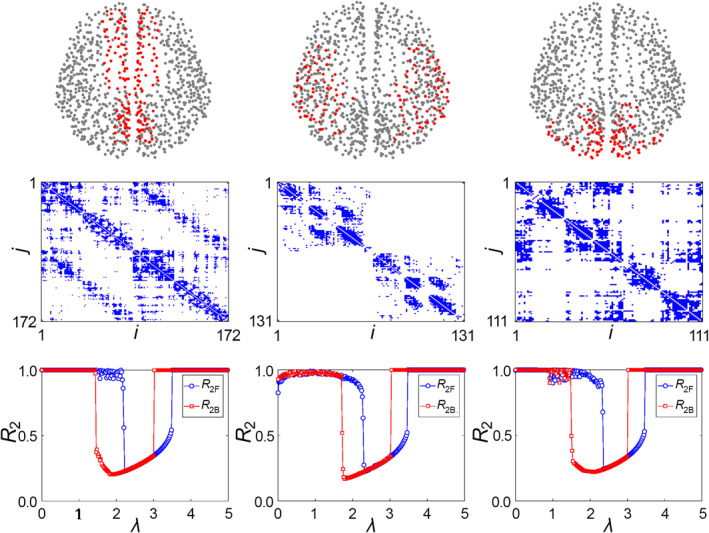
Synchronization of three typical cognitive subnetworks where the first, second, and third columns represent the cases of the medial default mode, ventral temporal association, and visual networks, respectively. The red points in the first row represent the nodes of the three cognitive networks, respectively, the three panels of the second row represent their connection matrix, respectively, and the three panels of the third row represent the dependence of their order parameter *R*_2_ on the coupling strength *λ*, respectively, where the blue line with “circles” represents the forward process and the red line with “squares” represents the backward process. Adapted from Ref. [48].

For this purpose, we notice that in general, a task is performed by only one specific cognitive subnetwork but not the whole brain network, that is, synchronization of one cognitive subnetwork. At the same time, it is also revealed that the activated brain network can be easily changed from one task to another one, indicating that a group of neurons needs to undergo an abrupt desynchronization from the correlated state associated to the first task to another different ordered state associated to the second task. To figure out the underlying mechanism, Wu et al. discussed the synchronization of three typical cognitive networks: the medial default mode, ventral temporal association, and visual networks [48]. Figure 5 shows their results where the red points in the first row represent the nodes of the three cognitive networks, respectively, the three panels of the second row represent their connection matrix, respectively, and the three panels of the third row represent the dependence of their order parameter *R*_2_ (see Ref. [48] for its definition) on the coupling strength *λ*, respectively. We see from the third row that all the three subnetworks display two consecutive explosive synchronization transitions, thus explaining the fast switch of brain network between different tasks.

In sum, various brain rhythms can be considered as different dynamics of network synchronization. As neurons are not independent each other but connected into a network, their unsynchronized activities will be randomized, that is, no rhythms. Once they are synchronized, the network activities will become regular and the corresponding EEG data will show rhythms, which is why we can observe different rhythms in EEG data.

## 3 UNIHEMISPHERIC SLEEP AND CHIMERA STATES

We may notice an interesting phenomenon that birds can sleep on trees for all night long but people cannot. The main reason is that during the sleep, a bird will keep one eye close and another open, in contrast to two closed eyes of human beings. This unique state that one cerebral hemisphere sleeps while another remains awake is named as unihemispheric sleep and has also been observed in other animals such as lizards, turtles and tortoises, and caiman. Especially, by taking time series of EEG, this phenomenon of unihemispheric sleep has been confirmed in aquatic mammals (cetaceans, eared seals, and manatees). For example, for the time series of EEG recorded from a bottlenose dolphin during unihemispheric sleep, its parieto‐occipital cortex shows synchronized high‐amplitude, low‐frequency EEG activity in the sleeping hemisphere and desynchronized low‐amplitude, high‐frequency EEG activity in the awake hemisphere [49]. These two different behaviors will alternatively change between the two hemispheres.

An intuitive explanation is that unihemispheric sleep can make animals to keep vigilance to external environmental, including human being, so that to provide self‐protection. A direct evidence is that when ducks sleep, the ones in the center are safely flanked by others and show bihemispherical sleep while others on the periphery of the group show unihemispherical sleep and orient their open eye watching for potential threats [50]. Then, a natural but interesting question is how about human being. Do we also have unihemispherical sleep? There is no answer for this question in a long time. Fortunately, Tamaki et al. discovered the answer in 2016 [51]. They considered the case that humans sleep in a novel environment and did observe the phenomenon of unihemispherical sleep of human being, named as the *first‐night effect* of human sleep.

The mechanism of unihemispheric sleep has been studied for about two decades as it was previously considered as a window to understand human brain, see reviews [12, 52, 53, 54, 55]. A main finding is that the mechanism of unihemispheric sleep can be explained by chimera state—a coexistence of one correlated part and another uncorrelated part. In details, this co‐existence was first discussed in a mathematical model in 2002 by Kuramoto and Battogtokh [56] and later named as chimera state by Abrams and Strogatz in 2004 [57]. In these two works, a key element is the nonlocal coupling, that is, neither the all‐to‐all coupling nor the nearest neighboring coupling. For example, the model of Abrams and Strogatz can be described as [57]

(10) $$\frac{\partial \phi }{\partial t}=\omega -\int_{-\pi }^{\pi }G\left(x-x^{\prime }\right)\sin\left[\phi \left(x,t\right)-\phi \left(x^{\prime },t\right)+\alpha \right]dx^{\prime }$$

where *φ*(*x, t*) represents the phase of oscillator and 0 ≤ *α* ≤ π/2 is a parameter. *G*(*x* − *x* ^′^) represents the coupling kernel and is taken as

(11) $$G\left(x\right)=\frac{1}{2\pi }\left(1+A\;\cos\;x\right)$$

where 0 ≤ *A* ≤ 1. It is a typical decaying function and thus represents a nonlocal coupling. By this kind of nonlocal coupling, a synchronized cluster will be remained once it is formed, thus leads to a coexistence of synchronized part and unsynchronized part.

Further, Abrams et al. presented a precisely solvable standard model of chimera state in 2008 [58]. This model consists of only two interacting groups of oscillators as follows

(12) $$\frac{d\theta_{i}^{\sigma }}{dt}=\omega +\sum_{\sigma^{\prime }=1}^{2}\frac{K_{\sigma \sigma^{\prime }}}{N_{\sigma^{\prime }}}\sum_{j=1}^{N_{\sigma^{\prime }}}\sin\left(\theta_{j}^{\sigma^{\prime }}-\theta_{i}^{\sigma }-\alpha \right)$$

where *σ* = 1, 2, *N*_*σ*_′ is the number of oscillators in group *σ*′. *K*_*σσ*_′ is the coupling strength and its value within each group is denoted as *K*_11_ = *K*_22_ = *μ* > 0, while that between the two groups is denoted as *K*_12_ = *K*_21_ = *v >* 0. For a set of randomly chosen initial conditions, Equation (12) will easily go to synchronization. However, very interesting, Equation (12) will show a chimera state when the initial conditions are chosen as one group approximately synchronized while the other group randomly [58]. This is a stabilized chimera state and will not show the feature of alternately changing the behaviors between the two hemispheres. To realize the alternating chimera state of birds and dolphins in sleep, Ma et al. introduced a periodic delay signal to describe the changing external environment [59]. Thus, Equation (12) becomes

(13) $$\frac{d\theta_{i}^{\sigma }}{dt}=\omega_{i}+\sum_{\sigma^{\prime }=1}^{2}\frac{K_{\sigma \sigma^{\prime }}}{N_{\sigma^{\prime }}}\sum_{j=1}^{N_{\sigma^{\prime }}}\sin\left(\theta_{j}^{\sigma^{\prime }}-\theta_{i}^{\sigma }-\alpha \right)+A\;\sin\;\Omega \left(t-\tau_{\sigma }\right)$$

where the term *A* sin Ω(*t* − *τ*_*σ*_) represents the external perturbation and *τ*_*σ*_ represents the time delay. Considering the fact that the two hemispheres should respond differently to the outside world, we may let *τ*_1_ = 0 and *τ*_2_ ≠ 0. The other parts of Equation (13) are considered as the same as that of Equation (12). Ma et al. found that the alternating chimera state can be observed [59], that is, the synchronization and unsynchronization periodically change between the two groups of Equation (13).

After that, the study of chimera state was extended to neural systems, see review [53]. For example, Omelchenko et al. considered a system of FitzHugh–Nagumo neurons with the nearest neighboring coupling and ring‐like topology [60]. The dynamics of their system can be described as follows

(14) $$\begin{array}{l} \varepsilon \frac{du_{k}}{dt}=u_{k}-\frac{u_{k}^{3}}{3}-v_{k}+\frac{\sigma }{2L}\sum_{j=k-L}^{k+L}\left[b_{uu}\left(u_{j}-u_{k}\right)+b_{uv}\left(v_{j}-v_{k}\right)\right],\\ \frac{dv_{k}}{dt}=u_{k}+a_{k}+\frac{\sigma }{2L}\sum_{j=k-L}^{k+L}\left[b_{vu}\left(u_{j}-u_{k}\right)+b_{vv}\left(v_{j}-v_{k}\right)\right],\begin{array}{cc} & k=1,2,\text{⋯} \end{array},N \end{array}$$

where *u*_*k*_ and *v*_*k*_ represent the activator and inhibitor variables, respectively. To observe chimera states of Equation (14), the coupling function is chosen as a rotational coupling matrix and can be introduced as follows

(15) $$B=\left(\begin{array}{cc} b_{uu} & b_{uv}\\ b_{vu} & b_{vv} \end{array}\right)=\left(\begin{array}{cc} \cos\;\phi & \sin\;\phi \\ -\sin\;\phi & \cos\;\phi \end{array}\right)$$

where *φ* represents the coupling phase and determines the property of coupling, that is, attractive or repulsive. This is a kind of cross‐coupling and has been widely used in the study of chimera states. Moreover, to reflect the feature of nonlocal coupling, the coupling strength *σ* is assumed to be a constant within the *L* nearest neighbors from both sides but zero otherwise. In detail, we let *r* = *L/N* be the parameter to represent the coupling radius.

**FIGURE 6:**
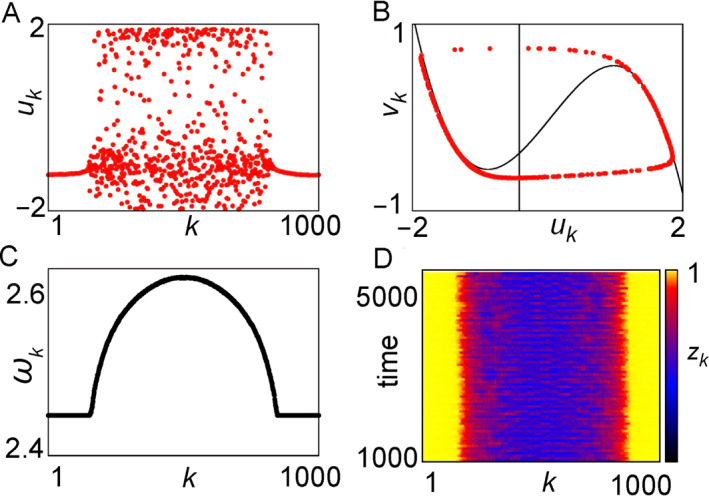
A typical chimera state for a coupled ring of the FHN neurons. (A) A coexistence of coherent and incoherent parts, (B) a snapshot in the (*u*_*k*_ *, v*_*k*_) plane (black lines denote the nullclines of the FHN system), (C) the mean phase velocities *ω*_*k*_, and (D) the local order parameter *Z*_*k*_. Reprinted with permission from Ref. [60]. FHN, FitzHugh–Nagumo.

Omelchenko et al. showed that it is possible for the system of Equation (14) to show both chimera states and other different behaviors [60]. For example, Figure 6 shows a typical chimera state, where (A) represents a coexistence of coherent and incoherent parts and (B) further confirms that the desynchronized part is distributed along the limit cycle.

On the other hand, the chimera state can be also characterized by the mean phase velocity of oscillators, $\omega_{k}=\frac{2\pi M_{k}}{\Delta T}$, with ∆*T* >> 1 and *M*_*k*_ being the total firing number of node‐*k* in ∆*T*. Figure 6C shows the results, where the constant part of *ω*_*k*_ represents the coherent part while the semicircle represents the incoherent part, confirming the coexistence of synchronized and unsynchronized parts. Further, the chimera state can be also represented by the local order parameter defined as

(16) $$Z_{k}=\left|\frac{1}{2\delta }\sum_{\left|j-k\right|\leq \delta }e^{i\theta_{j}}\right|,\begin{array}{cc} & k=1,2,\text{⋯},N \end{array}$$

with *θ*_*i*_(*t*) = arctan(*v*_*i*_ */u*_*i*_). Figure 6D shows the evolution of *Z*_*k*_ for *δ* = 25, where the yellow parts denote the coherent regions with *Z*_*k*_ = 1 while other parts denote incoherent parts with *Z*_*k*_ *<* 1.

Except the explanation of unihemispheric sleep, another application of chimera state is to explain the physical mechanism of brain functions. For examples, it was pointed out that chimera state is closely connected to various types of neuronal diseases such as epileptic seizures, Parkinsons disease, AD, schizophrenia and brain tumors [61]. It was also shown that chimera or chimera‐like states have strong connection to the bump behavior of neuronal networks associated with the mechanisms of working memory, head direction systems, and visual systems [62]. However, all these results are based on regular networks, while brain networks are complex networks. To extend the concept of chimera state to complex networks, Zhu et al. presented an approach in 2014 [63]. Their idea is to reorder all the oscillators by effective frequencies, otherwise the pattern will be messy as the complicated connections in a complex network will make an arbitrary pair of nodes be neighbors. Based on this reordering approach, many progresses on chimera states have been achieved in complex networks, including the brain networks, see reviews [12, 55].

**FIGURE 7:**
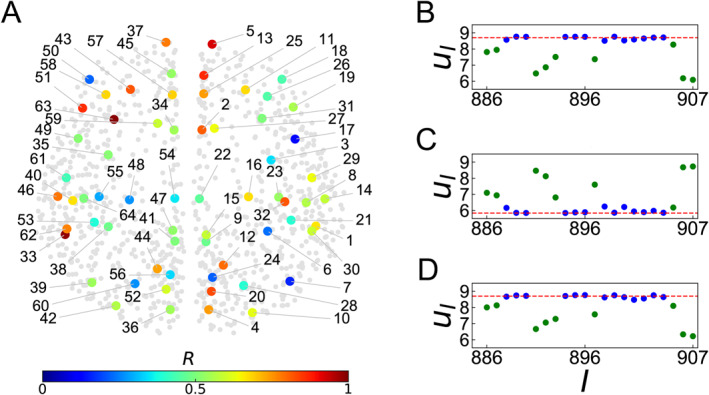
Multiscaled spatial chimera state. (A) A typical case of local representation of *R*, where different colors represent different values of *R* for all the 63 local brain subnetworks and the gray background points represent all the nodes of cerebral cortex network. (B–D) Show the snapshots of three arbitrary moments, respectively, for the cortical region 38 (lFUS) from (A). Adapted from Ref. [33].

Along this line, an interesting work was presented by Bansal et al. [8]. They found that the dynamical patterns of chimera states across cognitive systems and are closely related to structural variability, thus termed them as *cognitive chimera states*. Realizing that different cognitive functions are in fact implemented by cognitive systems but not the whole brain system, Huo et al. studied the chimera states for both the local and global networks and found that both levels can show chimera states, thus termed them as *spatial multi‐scaled chimera states* [33]. Surprisingly, they found that the chimera states between the local and global networks can be fundamentally different, that is, there are chimera states at the local level but no chimera states at the global level, indicating that the local chimera states of cognitive subnetworks take more important roles in brain functions. In details, Huo et al. discussed the brain network of Figure 1, which has 63 local brain subnetworks and each individual subnetwork has dozens of nodes. The nodes dynamics are chosen as the neural mass model of Equation (2). To quantify and distinguish different dynamical patterns in the 63 local brain subnetworks, an order parameter *R*, different from the local order parameter of Equation (16), can be defined by

(17) $${Re}^{i\phi }=\frac{1}{N_{j}}\sum_{I=1}^{N_{j}}e^{i\theta_{I}},$$

where *R* represents phase coherence, *θ* is the phase of oscillator, *ϕ* is the average phase, and *N*_*j*_ is the number of oscillators in the *j*th local brain subnetwork. The value of *R* will be unity for a fully synchronization, zero for disorder, and 0 < *R <* 1 for coherence of the oscillator population. *R* is a macroscopic quantity that characterizes the collective dynamics of the entire system. When *R* changes from zero to greater than zero, the system will undergo a phase transition from in‐coherence to coherence. The transition point is called as the critical point and the corresponding coupling is called as the critical coupling strength. In detail, the phase variable of *θ*_*I*_ can be calculated by *θ*_*I*_ = arctan(${\dot{v}}^{i}/{\dot{v}}^{e}$) for a system not having a well‐defined rotational center, that is, from the general idea of the curvature [64, 65]. Figure 7A represents the positions of the 63 local brain subnetworks in the whole brain network, where the gray background represents the nodes in all the 63 individual subnetworks, for visualization effect. Calculating *R* of Equation (17) for each individual subnetwork, Huo et al. found that the values of *R* are different from one subnetwork to another, see the different colors of Figure 7A. The average of *R* on all the local brain subnetwork will give the order parameter at the global level, which has a small *R*. Therefore, Figure 7A shows a multi‐scaled spatial chimera state. Moreover, to confirm the feature of chimera state at the local level, we take the cortical region 38 left fusiform gyrus (lFUS) from Figure 7A as an example, Figure 7B–D show its snapshots of three arbitrary moments, respectively. We see that the oscillators on the dashed lines represent the synchronized clusters while others unsynchronized, a typical chimera state.

In sum, both unihemispheric sleep and chimera states can be represented by local or partial synchronization. When a cluster of neurons are synchronized while the others are not, this local synchronization will perform a specific task, that is, a specific cognitive function. In this way, different synchronized clusters of neurons can perform different cognitive functions. This is the reason why human brain has powerful cognitive and memory functions.

## 4 LONG CONNECTIONS OF FUNCTIONAL BRAIN NETWORK AND REMOTE SYNCHRONIZATION

A remarkable difference between the brain structural network and brain functional network is that the former has denser local or short connections while the latter has denser global or long connections [31, 66, 67, 68]. An intuitive reason is that the structural brain network is a physical network where its connections in all possible pairs of nodes are measured from the number of fibers found by the tractography algorithm. This unique structure has two characteristic features: a huge number of neural connections and highly energy cost, that is, it has only about 2% of body mass but consumes 20% of the whole energy [2]. For the first feature, the cerebral cortex can put these huge number of connections into the limited volume of brain, resulting in the small‐world manner with community topology [9]. For the second feature, the brain network will try to save energy so that to reduce energy consumption. To satisfy this condition, the network structure will be rigorously controlled [69], which results in a community topology with less long‐distance connections and more local connections. Interestingly, these two contradictory features are naturally unified in the human brain network by a tradeoff between maximizing efficiency and minimizing wiring cost [70]. This economical tradeoff guarantees an efficient way to continuously process and transport information between functionally linked but spatially distributed regions [71].

In contrast to the brain structural network, the brain functional network comes from the Pearson correlation coefficients between the measured EEG time series [18], see Equation (1) for its calculation. For convenience, the functional brain network usually has the same set of nodes as that of the structural brain network but their connections are totally different. That is, the former has physical connections while the latter has correlation connections, that is, virtual links. In this way, the resulted functional brain network has completely different topology from its corresponding structural brain network [22]. Take the eight cognitive subnetworks of Figure 2 as an example. The nodes of these cognitive subnetworks are generally not completely composed of those neurons from the same local area but distributed on different locations of whole brain, indicating that they are chosen from remote places to implement a common task. In this way, the cognitive subnetworks will still work even when a local part of brain network is impaired or damaged. This is an intuitive reason for the functional connections to be different from the structural connections. Then, a natural question arises: what is the relationship between the long connections of functional network and the short connections of structural network, that is, the underlying mechanisms of the functional brain network. This is a key question to reveal the mechanisms of the functional organization of distant neural assemblies during various cognitive or pathological states [72].

**FIGURE 8:**
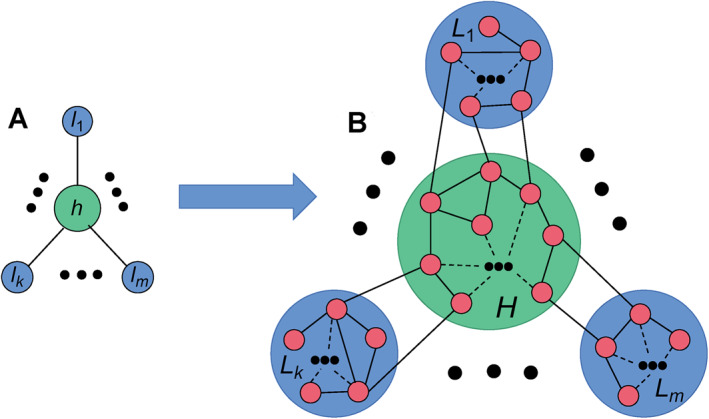
Schematic figure of RS. (A) Represents the schematic figure of star graph, where the central node‐*h* represents the hub node and the nodes‐*l*_1_–*l*_*m*_ represent the *m* peripheral nodes, respectively. (B) Represents the schematic figure of multi‐layered star‐like network, where the circle of *H* represents the central layer and the circles of *L*_1_–*L*_*m*_ represent the *m* leaf layers, respectively. Reproduced from Ref. [79] with permission. RS, remote synchronization.

To answer this question, a topic of RS is designed as a star graph by Bergner et al. in 2012 [73] and has gotten a great attention in recent years in the field of complex networks [12]. RS represents the phenomenon that two indirectly connected nodes will synchronize while the connected middle nodes will not synchronize [74, 75]. So far, it is found that RS is associated with topological symmetry where the nodes in remote locations of the network can swap their positions but do not influence the overall functioning of network [72, 76, 77, 78]. In 2012, this phenomenon is simplified into a paradigmatic model of star graph by Bergner et al., Figure 8A shows its schematic figure [79]. In this star graph, the central node‐*h* represents the hub node and the nodes‐*l*_1_−*l*_*m*_ represent the *m* peripheral nodes. Thus, RS denotes the phenomenon that there is no synchronization between the hub and leaf nodes but a synchronization among the *m* leaf nodes.

To measure RS, in contrast to the definition of order parameter of Equation (17) for a group of coupled oscillators, Bergner et al. introduced another way to calculate the order parameter for only two coupled oscillators as follows [73]

(18) $$r_{nm}=|\lim_{T\to \infty }\frac{1}{T}\int_{t}^{t+T}e^{i\left[\theta_{n}\left(t\right)-\theta_{m}\left(t\right)\right]}dt|$$

where *θ*_*n*_(*t*) represents the phase of oscillator *n* and *T* >> 1. In this framework, *r*_*nm*_ characterizes the phase synchronization between the two nodes *n* and *m*. *r*_*nm*_ will be unity when the phase difference *θ*_*n*_(*t*) − *θ*_*m*_(*t*) is fixed and zero when *θ*_*n*_(*t*) and *θ*_*m*_(*t*) are uncorrelated. Then, the order parameter among all the leaf nodes can be given as

(19) $$r^{\text{indirect}}=\frac{2}{l_{m}\left(l_{m}-1\right)}\sum_{n,m=1}^{l_{m}}r_{nm}$$

which is an average on all pairs of nodes in all the peripheral nodes. Comparing the two definitions of order parameter between Equations (17) and (19), we see that the synchronization by the former requires that all oscillators are synchronized while the latter only requires that the phase difference between each pair of nodes are bounded. That is, we will have a RS, provided that a bounded difference ∆*θ* exists. In the same way, we can give the order parameter for the links between the hub and leaf nodes as follows

(20) $$r^{\text{direct}}=\frac{1}{l_{m}}\sum_{m=1}^{l_{m}}r_{mh}$$

where *r*_*mh*_ runs over all the links between the hub and leaf nodes. *r* ^indirect^ (*r* ^direct^) will be in between 0 and 1, with 1 and 0 represent the phase synchronization and unsynchronization, respectively.

**FIGURE 9:**
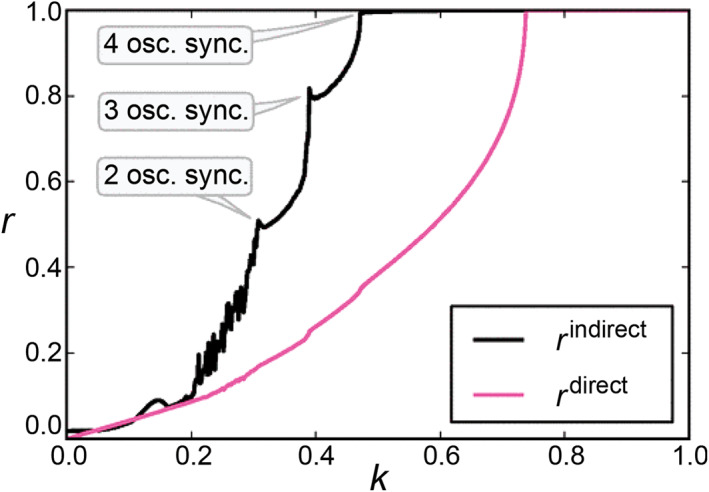
RS for the star graph of Figure 8A with *l_m_* = 4, where the “black” and “red” lines represent *r* ^indirect^ and *r* ^direct^, respectively. RS is the part with *r* ^indirect^ = 1 and *r* ^direct^ < 1, that is, 0.74 > *k >* 0.47. Reproduced from Ref. [73] with permission. RS, remote synchronization.

Specifically, Bergner et al. let the frequency of the hub node be approximately two and a half times of that of the leaf nodes [73]. They interestingly found that the unconnected leaf nodes will be synchronized, while the connected hub and leaf nodes will not be synchronized. That is, the hub will keep its own dynamics and just transmit the coupling for the leaf nodes. Figure 9 shows the dependence of these two order parameters *r* ^indirect^ and *r* ^direct^ on the coupling *k* for the case of *l*_*m*_ = 4. We see that the increase of *r* ^indirect^ is faster than that of *r* ^direct^, resulting in a hysteresis loop. RS can be expected in the range of this hysteresis loop. In detail, the leaf nodes will synchronize each other at *k* ≈ 0.47 while the synchronization between the hub and leaf nodes will be at *k* ≈ 0.74, indicating that the range of RS is 0.74 > *k >* 0.47.

After this paradigmatic work, the study of RS has been attracting more and more interest [54, 55, 80, 81, 82, 83, 84]. Especially, in brain networks, the patterns of coherent activity have been observed between cortical regions without connections [85]. Recently, Cao et al. extended the motif of Figure 8A into a more realistic situation with community structure [79], see Figure 8B for its schematic figure. It is in fact a model of multi‐layered star‐like network, where the circle of *H* represents the central layer and the circles of *L*_1_–*L*_*m*_ represent the *m* leaf layers, respectively, reflecting the characteristic feature of brain networks. Cao et al. found that this model shows the similar behavior as Figure 9, that is, a hysteresis loop, thus partially answered the existence of RS in brain networks [79].

**FIGURE 10:**
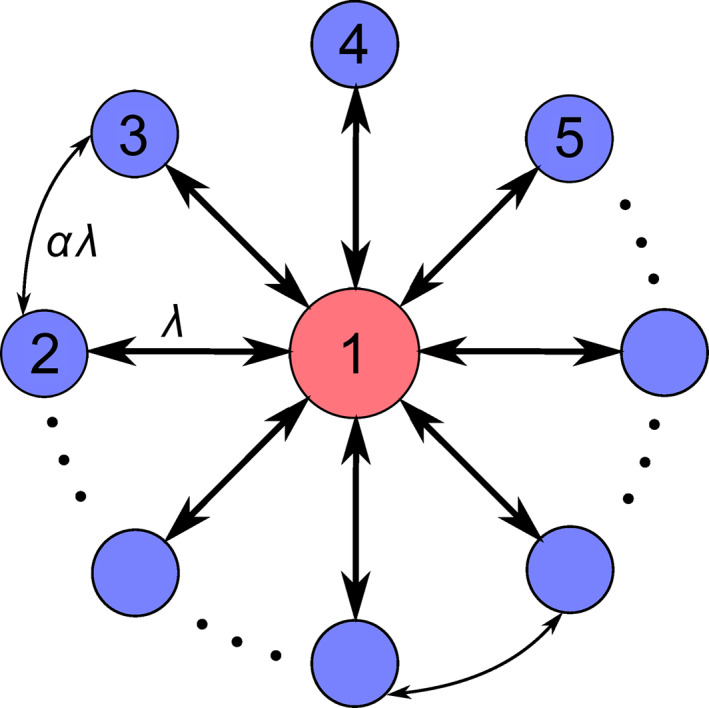
Schematic figure of the PRS model where the node‐1 represents the central hub node, the nodes‐2, 3, … represent the leaf nodes, and the links between leaf nodes represent the partial connections. *λ* represents the coupling between the hub and leaf nodes and *αλ* represents the coupling for the links between leaf nodes. Reproduced from Ref. [86]. PRS, *partial RS*.

Further, considering that each node in a brain network will be influenced by its neighboring nodes and then the neighbors’ neighbors and so on, Yang et al. extended the motif of Figure 8A into a star‐like motif where both the partial connections among the leaf nodes and the influence of the neighboring nodes of the leaf nodes are also considered [86]. In this model, we divide the leaf nodes into two parts: one part is the connected leaf nodes and the other is the unconnected leaf nodes. Figure 10 shows the schematic figure where the node‐1 represents the central hub node and the others represent the leaf nodes. We consider the couplings as bidirectional. As the connections between leaf nodes may exist in realistic data or come from the influence of the other parts of brain network, we would like to consider its coupling strength as *αλ*, with *λ* being the coupling between the hub and a leaf node. Figure 10 will return to Figure 8A when *α* = 0. Interestingly, Yang et al. found that RS will not emerge in all the leaf nodes but can appear only in the part of the unconnected leaf nodes, thus called it as *partial RS* (PRS) [86]. Moreover, they showed that the partial connections among leaf nodes favor PRS, indicating that it is easier for real brain networks to show RS than that of star graphs.

**FIGURE 11:**
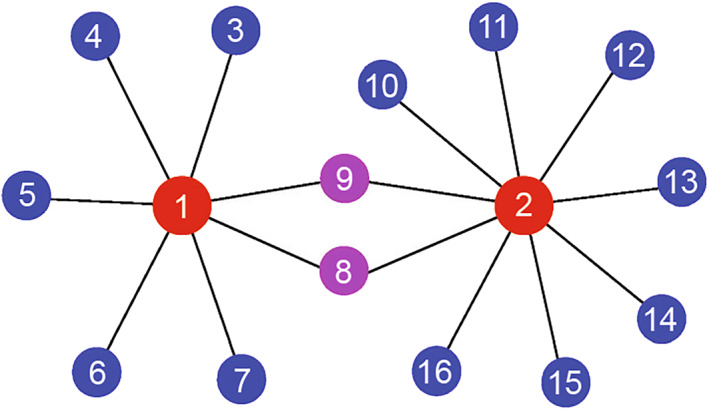
A schematic figure of multiple star‐like graphs connected by common leaf nodes, where the red, blue and pink nodes represent the hub, leaf and common leaf nodes, respectively. Reproduced from Ref. [81] with permission.

A shortage of above discussions is that the distance between two leaf nodes with RS is only two steps, which is still somehow different from the realistic functional brain networks with longer distance between synchronized leaf nodes. To go beyond the RS with two steps between leaf nodes, a framework of multiple star‐like graphs was presented by Kang et al. [81]. Figure 11 shows its schematic figure where two stars graphs are connected by the common leaf nodes 8 and 9. By this model, they showed that a RS between the leaf nodes of the two stars graph emerges [81], such as a RS between the leaf nodes 5 and 13. That is, the distance of RS has been extended to four steps. By this way, the distance of RS can be even extended to more steps, provided that we consider the framework of more connected star graphs.

In sum, we may conclude that RS is a possible way to generate the long connections of functional brain network from the nearest neighboring connections of structural brain network. This may be how a cognitive subnetwork is formed and is also the reason why brain networks have diversity of cognitive subnetworks, including the eight cognitive subnetworks in Figure 2. So far, the above studies are based on star or star‐like graphs and thus can be only considered as the primary steps toward the mechanism of long connections of functional brain network. Further progresses are expected on real functional brain network.

## 5 STRONGER DETECTION ABILITY OF HUMAN BRAIN TO WEAK SIGNALS AND REMOTE FIRING PROPAGATION

Various brain functions can be activated by the stimulation of external signals such as the visual cognitive subnetwork by lights and the auditory cognitive subnetwork by sounds. An empirical evidence is that human brain has an ability to detect weak signal when they pay attention enough, especially in an unfamiliar environment. Then, an important question is how the brain does. To figure out the mechanism, people studied this problem for a long time and realized that signal is detected by the activation of cognitive subnetworks of human brain. During these studies, an interesting finding is the *stochastic resonance*, which represents the higher detection ability of human brain to weak input signals under the assistance of noise [87, 88]. Later, it is found that noise can even be used to improve the temporal regularity of the bursting time series, named as *coherence resonance*, when there are no external signals [89, 90]. The main difference between stochastic resonance and coherence resonance is that the former focuses on the amplification of signal amplitude such as the signal‐to‐noise ratio while the latter deals with the temporal aspect of time series. Another difference is that stochastic resonance requires an external periodic driving while coherence resonance does not.

**FIGURE 12:**
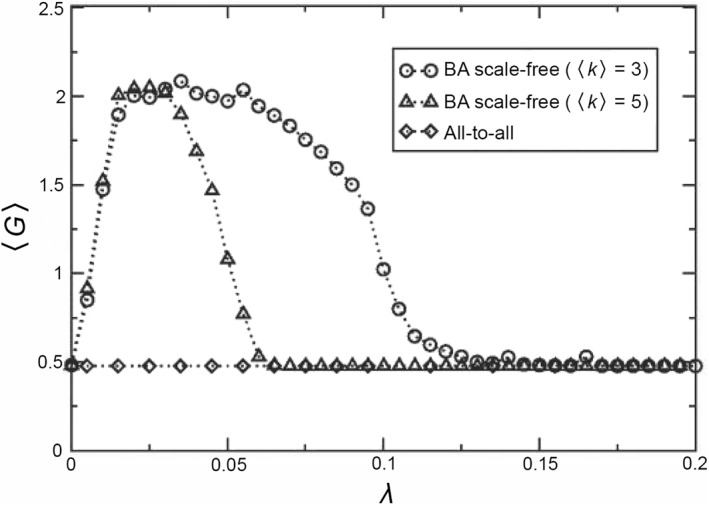
Dependence of the average amplification <G> on the coupling *λ* for both the scale‐free network and all‐to‐all network, where the lines with circles and triangles represent the results of BA network for the average degree 〈k〉=3 and 〈k〉=5, respectively, and the line with diamonds represent the results of all‐to‐all network. Reproduced from Ref. [91] with permission. BA, Barabasi–Albert.

Both stochastic resonance and coherence resonance do not discuss the influence of network structure. Considering that human brain is in fact a complex network, it is necessary to discuss how network structure influences the signal detection of brain network, especially for weak signals. In this aspect, some results have been obtained on artificial networks and it was revealed that the heterogeneity of scale‐free networks favors weak signals’ detection [91, 92, 93, 94]. For example, Acebron et al. considered a complex network where nodes are taken as overdamped bistable oscillators with a periodic signal as follows [91]

(21) $$\frac{{dx}_{i}}{dt}=x_{i}-x_{i}^{3}+\lambda \sum_{j=1}^{N}M_{ij}\left(x_{j}-x_{i}\right)+A\;\sin\;\omega t$$

where *M*_*ij*_ represents the connection matrix, *λ* is the coupling strength, and *A* sin *ωt* is the input signal with frequency *ω* and amplitude *A*. To measure the signal detection ability, Acebron et al. defined *G* = max(*x*_*i*_ */A*) as the amplification and then calculated the average amplification 〈*G*〉. They found that 〈*G*〉 will be seriously influenced by the network topology. For a heterogeneous network such as the Barabasi–Albert (BA) network with a scale‐free (power‐law) degree distribution [95], 〈*G*〉 will show a resonance behavior for the coupling strength *λ*, see Figure 12 where the lines with circles and triangles represent the results for the average degree 〈*k*〉 = 3 and 〈*k*〉 = 5, respectively. At the same time, they also made numerical simulations on the homogeneous all‐to‐all connectivity network but did not find this resonance phenomenon, see the line with diamonds in Figure 12. Their difference of behaviors is mainly come from the hubs of BA networks, that is, the heterogeneity of network degrees. Based on these results, the resonance behavior by heterogeneous topology is called *topological resonance*.

Above discussions are focused on artificial networks, then a natural question is how to extend the results to real brain networks, especially the individual cognitive subnetworks as they are responsible for the detection of signals. To address this problem, two kinds of experimental evidences have to be noticed. One is the weighted connections in cognitive subnetworks [5, 6]. Another is the balance of excitable and inhibitory neurons in brain networks [96, 97, 98, 99]. Based on these two aspects, a novel effect of heterogeneous couplings induced resonance is discovered and is named as *coupling resonance* [17, 100, 101]. It was shown that two kinds of heterogeneity can result in this coupling resonance, that is, one is the distributed couplings with mixed positive and negative parts and another is the nonidentity of oscillators. Especially, the coupling resonance has been confirmed in all the eight cognitive subnetworks of Figure 2, which can be used to explain the mechanism of detecting weak signals in brain networks [17].

In details, Deng et al. [17] recently considered the real cognitive subnetworks of Figure 2 and took the weights of connection matrices *W*_*ij*_ from the data of Refs. [5, 6]. It is well known that in brain networks, the population ratio between the excitable and inhibitory neurons is about 4:1 [96, 97, 98, 99]. However, each node in a cognitive subnetwork represents the neurons in a ROI, thus it is a mixed population of excitable and inhibitory neurons and cannot be simplified into an excitable or inhibitory node. In this sense, a reasonable way is to randomly consider part of the nodes as inhibitory and the others as excitable. That is, we may use a probability *p* to generate the inhibitory nodes and a probability 1 − *p* to generate the excitable nodes. According to Refs. [102, 103, 104], an inhibitory oscillator will have a negative coupling with its neighbors while an excitable oscillator will have a positive coupling with its neighbors. Thus, Equation (21) will be changed into

(22) $$\frac{{dx}_{i}}{dt}=x_{i}-x_{i}^{3}+\lambda_{i}\sum_{j=1}^{N}M_{ij}\left(x_{j}-x_{i}\right)+I\;\sin\left(\frac{2\pi }{T}t\right)$$

where *λ*_*i*_ will be positive for excitable oscillators and negative for inhibitory oscillators. For simplicity, all the values of *λ*_*i*_ are assumed to have the same amplitude *λ >* 0 but with *λ*_*i*_ = −*λ* for the inhibitory oscillators and *λ*_*i*_ = *λ* for the excitable oscillators.

To check the signal response, we follow Ref. [105] to introduce the spectral amplification factor *η* as

(23) $$\eta =\frac{4}{I^{2}}|<e^{i\frac{2\pi }{T}t}X\left(t\right){>}_{t}|$$

where $X\left(t\right)=\frac{1}{N}\sum_{i=1}^{N}x_{i}\left(t\right)$ denotes the average activity of the system.

**FIGURE 13:**
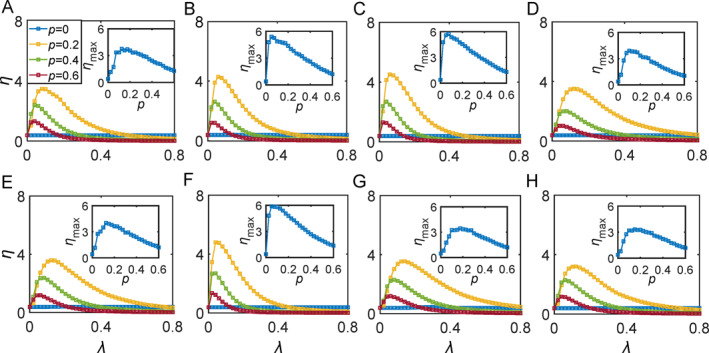
Dependence of the spectral amplification factor *η* on coupling resonance *λ* for the empirical cognitive subnetworks with *I* = 0.4, where the network topologies of the eight panels correspond to Figure 2A–H, respectively, that is, the cases of Aud, V, MS, VT, Att, mDm, CO, and FP cognitive subnetworks, respectively. The four lines in each panel denote the cases of *p* = 0, 0.2, 0.4, and 0.6, respectively, and the inset denotes the dependence of the maximum *η*_max_ on *p*. Reproduced from Ref. [17] with permission. Att, attention system; Aud, auditory; CO, cingulo‐opercular; FP, frontoparietal; mDm, medial default mode; V, visual; VT, ventral temporal association.

In numerical simulations, the case of *p* = 0, that is, no inhibitory oscillators, is firstly considered as a reference standard [17]. In this case, it is found that there is no coupling resonance of signal responses, indicating that the amplification of signal responses cannot be induced by only the distributed positive weights, that is, more other conditions are needed to generate the coupling resonance. The lines of *p* = 0 in Figure 13A–H show the results, where (A–H) denote the cases of Aud, V, MS, VT, Att, mDm, CO, and FP cognitive subnetworks of Figure 2A–H, respectively. We see that there is no coupling resonance of signal responses in all of them. Then, we discuss the cases of *p >* 0. Figure 13A–H show the results for *p* = 0.2, 0.4, and 0.6, respectively. We find that all of them are bell‐shaped, indicating the coupling resonance of signal responses. Thus, the necessary condition for coupling resonance is the mixed positive and negative couplings.

Further, we see from Figure 13A–H that there is an optimal *λ* for each case of *p >* 0, where *η* reaches its maximum *η*_max_. We also find that the value of *η*_max_ is different for both different *p* and different cognitive subnetworks, implying that we may use the maximum *η*_max_ to reflect the differences between the cognitive subnetworks. For this purpose, the dependence of *η*_max_ on the parameter *p* is calculated, see the results shown in the insets of Figure 13A–H. We see that the values of *η*_max_ are larger in the panels of Figure 13B,C,F but smaller in the other five panels, indicating that different cognitive subnetworks have different signal detection ability.

In sum, both Equations (21) and (22) discuss the case that the external signal has been received by all nodes of network. However, this is not the only aspect of detecting signals in brain network. Another aspect is how signals are propagated from one neuron to another. In this aspect, an efficient way is that we let one node be the source node with signals and study its spreading in the network [83, 84, 106]. As the number of neurons in human brain is huge, it is difficult to study the firing propagation. Therefore, we may try a smaller neural network. In this sense, we here take the network of *Caenorhabditis elegans (C. elegans)* as an example, which has only 277 neurons [107]. For this purpose, Shen et al. considered this *C. elegans* network and let the nodes dynamics be represented by the Hindmarsh–Rose model as follows [83]

$$\frac{dx_{i}}{dt}=y_{i}+bx_{i}^{2}-ax_{i}^{3}-z_{i}+I_{ext}+\lambda \sum_{j=1}^{N}A_{ij}\left(x_{j}-x_{i}\right)$$

$$\frac{dy_{i}}{dt}=c-dx_{i}^{2}-y_{i}$$

(24) $$\frac{dz_{i}}{dt}=r\left[e\left(x_{i}-x_{0}\right)-z_{i}\right]$$

where *A*_*ij*_ denotes the connection matrix of *C. elegans* network from Ref. [107] and the parameters are taken from Ref. [108]. In this set of parameters, Equation (24) will be inactivated. We randomly choose one node as the source node‐*s* to receive an extra external signal *I*_*s*_ so that it can be activated. Then, we study how the firings of this source node‐s is spread on the network. In detail, the first equation of Equation (24) will be replaced by

(25) $$\frac{dx_{s}}{dt}=y_{s}+bx_{s}^{2}-ax_{s}^{3}-z_{s}+I_{ext}+I_{s}+\lambda \sum_{j=1}^{N}A_{sj}\left(x_{j}-x_{s}\right)$$

for the source node‐*s* and remains unchanged for all the other nodes, that is, the target nodes.

**FIGURE 14:**
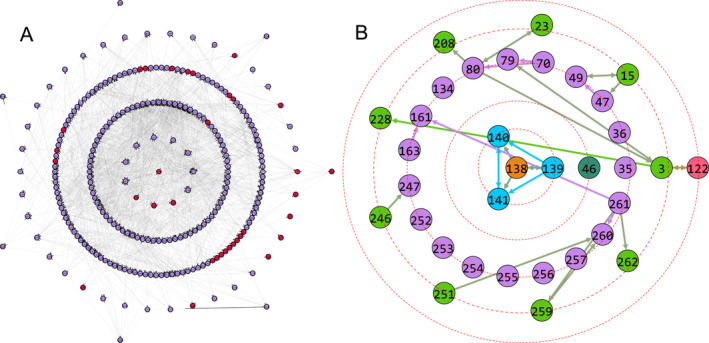
A typical example of RFP by choosing the node‐138 as the source node for the neural network of *C. elegans*. (A) The network topology of neural network of *C. elegans* with the source node‐138 at the center, where the red nodes represent the activated nodes by the source node‐138. (B) The network topology of only the activated nodes and the links among them in (A), where the different colors of nodes in different circles are for eyes’ guide. Reproduced from Ref. [83] with permission. RFP, remote firing propagation.

It is found that depending on the chosen source node‐*s*, the firing spreading may be either local or global. Except these normal propagations of signals, Shen et al. found an interesting phenomenon that the firings can be propagated to distant nodes even without the activation of intermediate nodes, thus named it as *remote firing propagation* (RFP) [83]. Figure 14A shows such an example of RFP by the source node‐138 where the center represents the source node‐138, the nodes on the first circle represent the nearest neighboring nodes of the source node‐138, and that on the second circle represent the nearest neighbors’ neighbors and so on. All the activated nodes by the source node‐138 are represented by the red nodes. We see from Figure 14A that the connections are very dense and most of them has no relationship with the activated nodes, thus it is maybe better to remove them. In this sense, we keep only the red nodes and those links among them in Figure 14A but remove all the other nodes and their links, resulting in Figure 14B, which is convenient for us to figure out the features of RFP. Further, we use different colors to distinguish the nodes of Figure 14B on different circles. We see from Figure 14B that the source node‐138 is only connected to the three nodes 139–141 of the first circle and thus they form a core of four connected nodes, but they are not directly connected to all the other nodes, indicating an indirect connection between them. Moreover, some of them are even isolated, such as the nodes 46, 35, and 253.

**FIGURE 15:**
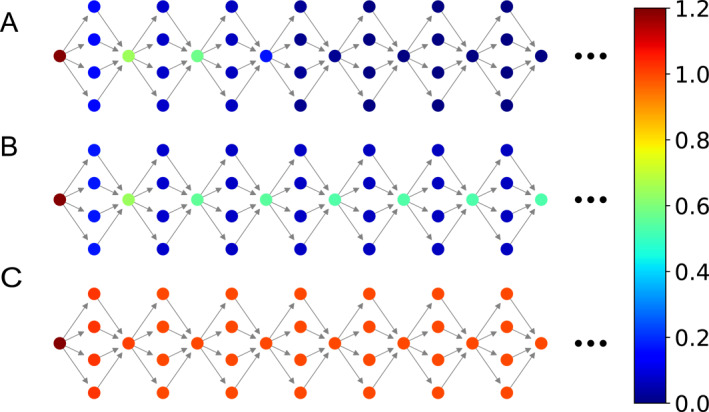
Schematic illustration of RFP by the heterogeneous chain model where the first red node is the source node and all the others are the target nodes. The arrows represent the directions of signal propagation. (A–C) Represent the results of finite propagation, remote propagation, and infinite propagation, respectively, with *λ* = 0.19, 0.2, and 0.3, respectively. Reproduced from Ref. [84] with permission. RFP, remote firing propagation.

The mechanism of RFP may be also understood by a double‐well system, which is a simplified toy model to describe the node dynamics of the brain network, as it ignores the complicated aspects of neural models but keep the most important feature of two states, that is, firing or quiescence, remain unchanged. When the network topology is chosen as the empirical brain network of Figure 1A, Wang et al. showed that RFP can be observed [84]. To explain the mechanism of RFP, a heterogeneous chain model was designed where the first red node is set as the source node and all the others are set as the target nodes, see Figure 15 for its topology. The arrows represent the directions of signal propagation. It was found that the signal can be either propagated or not, depending on the value of coupling strength *λ*. Figure 15A–C show the results for three typical cases of *λ* = 0.19, 0.2, and 0.3, respectively. We see that the signal cannot be propagated in Figure 15A but can in Figure 15C. While Figure 15B represents a typical FRP where the firing hub nodes are separated by the unfired leaf nodes. The reason is that in Figure 15B, the coupling strength *λ* is not large enough to induce the firings of leaf nodes but the coupling accumulation from all the leaf nodes is large enough to induce the firings of hub nodes. Therefore, the condition for RFP is that for a source node‐*s*, the coupling accumulation of its (*n* + 1)th step neighbors is larger than the critical coupling while the coupling accumulation of all the directly connected *n*th step neighbors is smaller than the critical coupling.

So far, the study of RFP is still in its primary stage, but it opens a new window to help us to understand the emergence of cognitive brain networks.

In sum, except the noise enhanced signal detection in previous studies, current studies show that it is also possible to enhance signal detection by topological resonance and coupling resonance. For the former, the scale‐free network is a typical example where the hub nodes with larger degrees will receive more couplings from their neighboring nodes and thus will be easier to be activated, resulting in the effect of topological resonance. While for the later, the coexistence of positive and negative couplings represents the coexistence of excitable and inhibitory neurons. This coexistence will make an oscillator to jump between the two wells and thus induce a big oscillation behavior, resulting in the effect of coupling resonance.

## 6 PATTERNS OF DEMENTIA AND EIGEN‐MICROSTATE ANALYSIS

To support diverse cognitive task performances, the human brain dynamically reconfigures its functional organization. During this process, the most important features of human brain are its segregation and integration. It is pointed out that both integration and segregation are closely associated with general cognitive ability, memory, and crystallized intelligence and processing speed [109]. So far, the most studied is the resting state of brain networks, which is independent from specific task demands. It is revealed that brain is active even in the absence of task, where most of the brain’s energy is consumed by intrinsic functional activity [110, 111]. In resting state, there is no cognitive tasks but the functional organization of brain can be seen through patterns of ongoing spontaneous activity. For example, based solely on spontaneous activity, Fox et al. identified a bilateral dorsal attention system and a right‐lateralized ventral attention system [111]. A natural question is why spontaneous activities can reflect task‐related activity patterns. One possibility is that the brain’s functional organization at rest can mirror relevant task‐induced activity patterns and thus predict task performance [112, 113]. Therefore, spontaneous activities of the human brain can be considered as a window to reveal the mechanisms of brain functions.

Based on this window, it might be possible to go a further step to study the relationship between the functional and structural brain networks. In general, our brain is not always in the normal state but may also show abnormal states such as AD, Parkinson’s disease, and aging. These patterns of dementia will fall into dissociated but dispersed brain networks, thus studying the relationship between structural and functional brain networks may provide helps to sustain healthy. On the other hand, it was suggested that resting brains usually operate near a critical state so that brains can quickly explore and switch in state space [11, 112, 114, 115, 116]. Thus, the resting state networks is the right system for our purpose. By this system, it has been demonstrated that the approach of eigenmode analysis can bridge the brain functional and structural networks, as the eigenmodes are consistence among healthy subjects, but show significant difference in impaired brains [117]. That is, eigenmode analysis can be used to distinguish healthy brain function and pathophysiology of disease.

Eigenmode analysis is not based on the pairwise interaction, but on the level of eigen‐spectra of network. In fact, this approach is not strange for us but has a longer history [118, 119]. For example, in the case of a violin string, it is in direct analogy to Fourier analysis. Of course, network dynamics is more complicated than that of a violin string, in which each spatial pattern has a unique frequency [120]. On the other hand, eigenfunctions are also very important for classical mechanics, for example, standing waves in continuous media are eigenfunctions. And in quantum mechanics, the eigenfunctions of the Schrodinger wave equation can be used to describe the probability cloud of the electron’s orbit around the nucleus. Its main idea is that the emergence of dynamical patterns is closely related to the activation of intrinsic eigenmodes of network. Inversely, individual eigenmodes will gradually emerge in system dynamics when we decrease the coupling, indicating a good predictor of functional eigenvectors. Just as we have discussed in Section 2 of network synchronization, different synchronization clusters will emerge with the decrease of coupling strength [46].

To confirm the relationship between the eigenvectors of connectome Laplacian and that of functional eigenvectors [117, 121, 122, 123], Raj et al. studied the patterns of dementia [122], which is a serious disease and can affect 25 million people worldwide, including 30%–70% AD and 10% frontotemporal dementia (FTD). They first obtained the brain networks of 14 young healthy volunteers by diffusion MRI scans and whole brain tractography. Then, they use a linear diffusion process to model dementia progression as follow

(26) $$\frac{dx_{1}}{dt}=\beta c_{1,2}\left(x_{2}-x_{1}\right)$$

where *β* is the coupling strength, *c*_1,2_ the inter‐region connection strength, and *x*_2_ − *x*_1_ the concentration difference of the disease factor between the regions of *x*_2_ and *x*_1_. Going back to brain network, the disease factor *x*_*i*_ will be represented by the vector *x*(*t*) and the connection strength *c*_*i,j*_ will be represented by the connection matrix. Thus, Equation (26) will become

(27) $$\frac{dx\left(t\right)}{dt}=-\beta \mathrm{Hx}\left(t\right)$$

where *H* represents the Laplacian matrix, with *H*_*i*,*j*_ = −*c*_*i*,*j*_ for *c*_*i*,*j*_ ≠ 0, $H_{i,j}=\sum_{j^{\prime }}c_{i,j^{\prime }}$ for *i* = *j*, and *H*_*i,j*_ = 0 otherwise. The eigenvalues *λ*_*i*_ of *H* will be in the range of [0, 1] [122], which has a single 0 eigenvalue and a small number of near‐zero eigenvalues.

**FIGURE 16:**
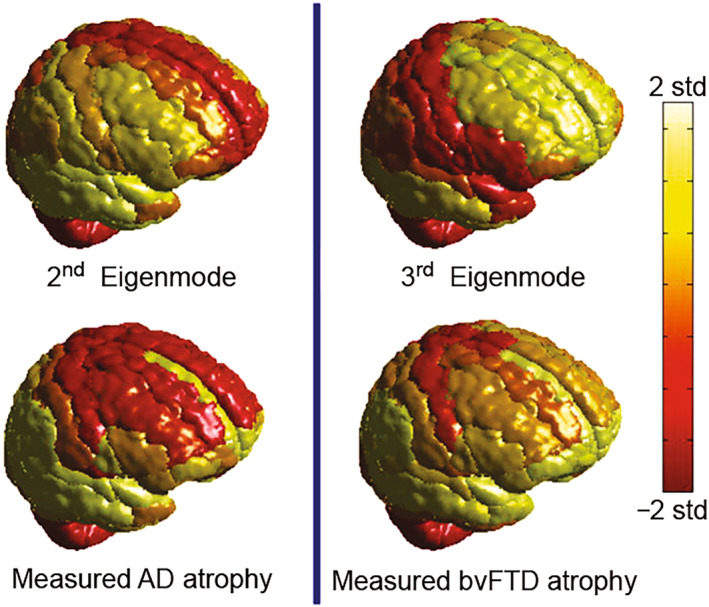
The two panels of the first row represent the second and third eigenmodes of the healthy networks, respectively, and the two panels of the second row represent the atrophy patterns of AD and bvFTD, respectively. Both eigenmode values and atrophy are converted into *z*‐scores and mapped to the range shown by the colorbar. Reproduced from Ref. [122] with permission. AD, Alzheimer’s disease; bvFTD, behavioral variant of FTD.

By Equation (27), Raj et al. calculated the cortical atrophy and found that only the small eigenmodes will be remained, while most large eigenmodes will be quickly decayed [122]. At the same time, they calculated the first three eigenmodes of *H*, which is assumed to predict the dementia atrophy patterns. Then, by comparing the measured atrophy of dementia patients with the predicted patterns, they found strong evidence that the healthy networks eigenmodes are one‐to‐one correspondence with the atrophy patterns of normal aging, AD, and behavioral variant of FTD (bvFTD). Figure 16 shows their results of the second and third eigenmodes, where the top row represents the second and third eigenmodes, respectively, and bottom row represents the data of AD and bvFT, respectively. The first eigenmode with *λ*_1_ = 0 is not shown as it is trivial and just varies simply according to region size. Comparing the top row of Figure 16 with the bottom row, we see that they are in good agreement with each other, confirming that the patterns of dementia can be predicted by the eigenmodes of connectome Laplacian.

Further, Chen et al. recently discussed the reconstruction of functional connectivity based on the five leading basic modes and found that the identification accuracy reached surprisingly 92% [124]. They applied the eigen‐microstate analysis of Refs. [125, 126] to the dataset of Human Connectome Project (HCP) to figure out its basic modes of brain activity. Their method consists of the following steps:

1. Firstly, we extract the time courses of all the network nodes and transform them into *z*‐score values with zero mean and unit variance over time. Using these data, we obtain a *N* × *M* matrix A, with *N* being the number of nodes and *M* being the number of time points in the time series. Each column, A_*t*_, denotes a microstate of brain activity at time *t.*
2. Secondly, we calculate the covariance matrix between microstates as C = A^*T*^ A and then compute its eigenvectors by
(28) $${\text{Cv}}_{i}=\lambda_{i}v_{i}$$
  wherein *λ*_*i*_ is the *i*th eigenvalue of the matrix C, and *v*_*i*_ is the corresponding eigenvector. Each eigen‐microstate can be obtained as the weighted sum of the origin microstates as follows [125, 126]
(29) $$E_{i}=Av_{i}=\sum_{t=1}^{M}A_{t}v_{ti}$$
  where *v*_*ti*_ represents the *t*th element of eigenvector *v*_*i*_ and quantifies the contribution of *t*th microstate to the *i*th eigenvector.
3. Thirdly, we use the singular value decomposition to obtain the eigen‐microstates [125].
  By these steps, Chen et al. identified five leading basic modes of spontaneous activity [124]. They found that the weights of these basic modes decreased quickly with the increase of ranking, indicating that the other models are not so important. They thus concluded that this is the reason why a small number of Laplacian eigenmodes can be used as the basis function to successfully reconstruct the functional connectivity matrices.

Different from the above eigen‐microstate analysis, Wang et al. presented an alternative approach to calculate eigenmodes [109, 116]. Instead of the covariance matrix C, Wang et al. directly calculates the eigenmodes of structural brain networks. Their method consists of the following steps [116]:

1. Firstly, we calculate the Laplace matrix *H* = *D* − *A*, where *A* = {*A*_*ij*_} represents the structural connection matrix of brain network and *D* is a diagonal matrix with $D_{ij}=\sum_{j^{\prime }}A_{ij^{\prime }}$ for *i* = *j*, and *D*_*ij*_ = 0 otherwise. Further, we let *λ* and *V* be the structural eigenvalues and eigenvectors, respectively. Then, we decompose the Laplace matrix *H* as *H* = *V λV* ^*T*^. These structural eigenmodes will be sorted according to the eigenvalues in ascending order [123].
2. Secondly, we construct the matrix *C* of functional connection networks from the fMRI data and decompose it as *C* = *U* Λ*U* ^*T*^, where Λ and *U* represent the functional eigenvalues and eigenvectors in descending order, respectively.
3. Thirdly, we use the structural eigenvectors *V* as a set of orthogonal bases to describe the functional eigenvectors *U.* Thus, we have
(30) $$U_{i}=\sum_{j=1}^{N}m_{ij}V_{j},\left(i=1,2,\text{…},N\right)$$
  where $m_{ij}=V_{j}^{T}U_{i}$ and $\sum_{j=1}^{N}{\left(m_{ij}\right)}^{2}=1$. Then, we can measure the contribution of the *j*th structural eigenmode to the functional network by
(31) $$S_{c}\left(j\right)=\sum_{i=1}^{N}{\left(\Lambda_{i}m_{ij}\right)}^{2}$$

By the using of this eigenmode analysis, Wang et al. investigated the joint contribution of hierarchical modular network to brain functional diversity [109, 116]. By examining the dependence of functional patterns on the inherent structural modes, they confirmed that the activation of structural modes can be measured by the contribution *S*_*c*_(*j*) over all the functional modes.

Along this line, Huo et al. recently extended this eigenmode analysis to nonlinear diffusion model, that is, the neural mass oscillators of Equation (2), and made a connection between the eigenmodes and chimera states [127]. Very interesting, they found that the dynamical emergence of lower eigenmodes is in fact a condensation, similar to the famous *Bose–Einstein condensation*. Different from all the previous studies, Huo et al. considered the direct relationship between structural brain network and its dynamics and then try to figure out its local phase space for brain activation, thus this idea might be extended to other networks and other kinds of oscillators.

A key feature of Ref. [127] is the time delay in Equation (2), which comes from the finite conduction speeds in brain networks. As time delays should be based on the distances between brain regions and choice of a biologically meaningful conduction speed, the time delay *τ*_*ij*_ between the nodes *i* and *j* can be obtained by

(32) $$\tau_{ij}=\frac{d_{ij}}{v}$$

where *v* is the conduction speed and *d*_*ij*_ is the distance between the nodes *i* and *j*. The distances *d*_*ij*_ can be found from Ref. [6] and most of them are smaller than 100 mm. For the conduction speed *v*, it may be different for different brain networks. For the rabbit brain, the axonal conduction velocities are found to be 1 m/s for ipsilateral cortico‐cortical connections and 1.5 m/s for callosal cortico‐cortical fibers [128, 129]. While for human brain, the conduction velocity is approximately 1.7 m/s for myelinated and 0.1 m/s for unmyelinated fibers [130]. However, in contrast to Ref. [130], Ref. [131] shows that the conduction velocity through unmyelinated axonal fibers is on the order of 1 m/s. Anyway, the conduction velocity is complicated, that is, depending on agents.

**FIGURE 17:**
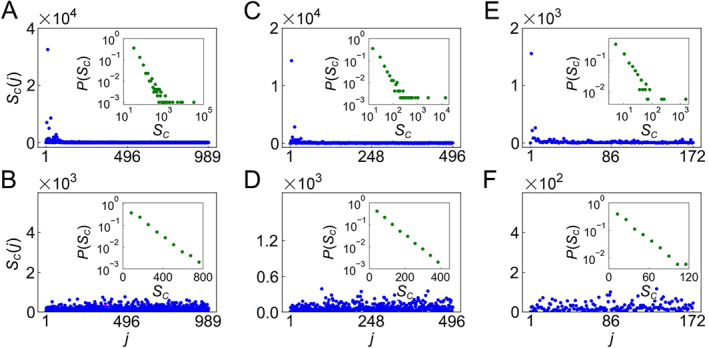
The values of *S*_*c*_(*j*) and its distributions of *P*(*S*_*c*_), where the first and second rows represent the cases of typical condensation and non‐condensation, respectively. In the first row, the dot in the red circle is much larger than others, marking the condensation, and the red circle is for visualization. (A, B) represent the case of global brain network, (C, D) the network of right hemisphere, and (E, F) the cognitive network of medial default mode. Reproduced from Ref. [127].

By considering the influences of both the coupling strength *c* and time delay *τ* of Equation (2), we now discuss how the eigen‐modes of a functional brain network show a condensation. We obtain *S*_*c*_(*j*) by following Equation (31). According to Ref. [116], we artificially set the contribution from the trivial eigenmode with *λ*_1_ = 0 as *S*_*c*_(1) = 0.1 to avoid its too large contribution. By this way, we find that *S*_*c*_(*j*) will seriously depend on the parameters *c* and *τ*. Especially, *S*_*c*_(*j*) will show a condensation when *c* and *τ* are matched well. Figure 17A,B show two such examples of Equation (2), with (*c* = 5.3*, τ* = 19) in (A) and (*c* = 0.3*, τ* = 9) in (B). In Figure 17A, we can see a dominant *S*_*c*_(12) (represented by the red circle), indicating that the functional network is mainly activated by the 12th structural eigenmode, that is, a *condensation* on the 12th structural eigenmode. While in Figure 17B, we cannot see such a dominant *S*_*c*_(*j*), that is, no condensation. In the same way, we also observe condensation at both the level of hemispheres (see Figure 17C,D for the right hemisphere) and the level of cognitive networks (see Figure 17E,F for the cognitive network of medial default mode), thus condensation is one reason for chimera states or brain functions.

**FIGURE 18:**
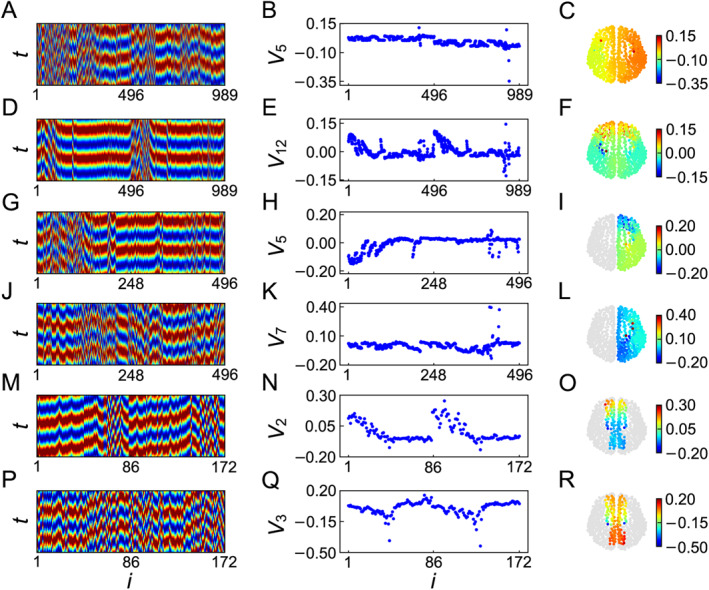
Six typical condensations with the lower condensed eigen‐modes where (A–C) and (D–F) represent the case of global brain network, (G–I) and (J–L) represent the case of right hemisphere, and (M–O) and (P–R) the case of the cognitive network of medial default mode. The left column represents the spatia‐temporal patterns from Equation (2), the middle column represents the condensed structural eigenmodes, and the right column represents the projection of the structural eigenmodes of the middle column on the cerebral cortex for visualization. Reproduced from Ref. [127] with permission.

For visualization, it is maybe interesting to show a few condensed structural eigenmodes. For this purpose, six typical condensations are given in Figure 18 where the first two rows, that is, (A–C) and (D–F), denote the case of global brain network, the second two rows, that is, (G–I) and (J–L), denote the case of the right hemisphere, and the third two rows, that is, (M–O) and (P–R), denote the case of the cognitive network of medial default mode. The left column represents the evolution of dynamics from Equation (2), that is, spatiotemporal patterns, the middle column represents the structural eigenmodes of condensation, and the right column represents the projection of the structural eigenmodes of the middle column on the cerebral cortex for visualization. It is clear that all the spatiotemporal patterns in the left column are coexistence of correlated and uncorrelated clusters, that is, chimera states, and all the condensed structural eigenmodes *V*_*j*_ in the middle column are much less than the system size *N*, that is, limited to the smaller eigenmodes with *j* ≤ 12, confirming from the aspect of condensation that only the lower structural eigenmodes are nontrivial.

In sum, the eigen‐microstate analysis is a kind of spectra analysis, in contrast to the pairwise interaction in the studies of both synchronization and partial synchronization. This eigen‐microstate analysis takes the network as a whole and thus can reveal more information than the pairwise interaction, such as the patterns of dementia being condensations of specific eigenmodes.

## 7 DISCUSSIONS AND OUTLOOKS

So far, we have briefly reviewed the main results of exploring the physical mechanisms of human brain functions from the aspects of network synchronization, partial synchronization, and related firing propagation. However, these aspects are not all modes or angles but only some. A common point among them is that all of them can be applied to understand brain. For examples, the network synchronization can be used to explain not only the abnormal synchronization of epileptic seizure [34] but also the various rhythms in brain, such as the delta (0−4 Hz), theta (4−7 Hz), alpha (8−13 Hz), beta (13−30 Hz), and gamma (30−100 Hz) rhythms revealed in EEG data [23]. They provide the initial windows for us to understand our human brain and can also help us to check our health status, that is, whether we are in sub‐health status. The partial synchronization includes both the chimera states and RS, which are closely related to the memory and cognitive brain functions [72]. As each individual cognitive function is executed by only one or a few subnetworks but not the whole brain network, the study of partial synchronization provides us deep insights into the mechanisms of brain functions such as the *first‐night effect* of human sleep [51] and the denser long connections in brain functional network [67], paved a way to the study of brain. A further study on multi‐scaled spatial chimera state [33] may help us to understand the fast transition between different cognitive tasks but is still an open question. While for the firing propagation, it can show how synchronization emerges in brain, which can be influenced by both the network topology [91] and the mixture of excitable and inhibitory neurons [17]. Even more, it was pointed out that the ongoing spontaneous firing patterns in the resting state are not random but show a memory to task‐related activity patterns [112, 113], which may be used to predict task performance.

On the other hand, all the above discussions are based on neural models. However, in our brains, there are not only neurons but also glial cells which populate both peripheral and central nervous systems (CNS). In a brain, neurons make up almost half of the cells within the brain but are outnumbered by glial cells [132]. Glial cells mainly constitute astrocytes, microglia, and oligodendrocytes where astrocytes account for 19%–40%, microglia constitute 10%, oligodendrocytes constitute 45%–75%, and the remaining cells are NG2 cells [133]. Glial cells were previously regarded as providing structural and nutritional supports to neurons. In recent years, it has been recognized that glial cells are involved in the development of the CNS under normal physiological conditions. Especially, glial cells take important role in neurobiology and prion neuropathology [134], including both beneficial and detrimental effects, such as in the aspects of ischemic stroke [133], schizophrenia [135], AD [136], glaucoma [137], and protein aggregation [138]. Glial cells, and in particular microglia, can function as immune cells [139]. Thus, studying the switch between detrimental and protective phenotypes represents a promising direction to find new therapeutic approaches [140]. Recently, Wang et al. presented a model of neuron‐astrocyte network to study the roles of astrocytes in regulating the cluster synchronization behaviors and found a phenomenon of breathing cluster [141], where part of neurons on the network form a synchronized cluster, while the remaining neurons are kept as desynchronized. Very interesting, they showed that the cluster is switching between the synchrony and asynchrony states in an intermittent fashion, partially revealing the mechanism of the state‐switching of the neocortex. More progresses are expected in this direction.

**FIGURE 19:**
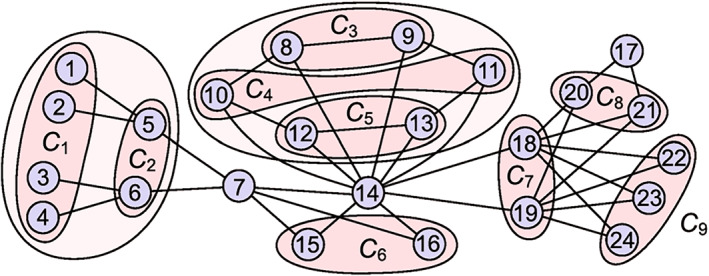
Cluster synchronization from symmetric connections of nodes, where *C*_1_–*C*_9_ are the synchronized clusters with strict symmetry in each cluster. Reproduced from Ref. [144] with permission.

Although these great progresses, it is still far away from the complete understanding of brain functioning. To speed up further studies in the future, we suggest the following directions:

1. **Remote synchronized clusters from asymmetric connections of nodes**. Except the discussed RS in Section 4, another approach to study the remote connections of functional brain network is cluster synchronization. In this state, the oscillators in a particular group of network will be synchronized and the behaviors of different synchronized groups will be different. It has been shown that the symmetry of network topology takes a key role in the emergence of cluster synchronization [142, 143, 144, 145, 146, 147, 148], see review [149] for details. For example, Figure 19 shows all the groups of symmetry clusters in a cluster synchronization pattern for a 24‐node network [144], where *C*_1_–*C*_9_ are the synchronized clusters with strict symmetry in each cluster.
  Except the feature of symmetric connections in each group of *C*_1_–*C*_9_ in Figure 19, another feature is that each individual node in the same group of cluster synchronization receives the same total input couplings from other nodes. However, these two features are very strict and generally cannot be satisfied by the nodes of human brain structural networks. In principle, the first feature of symmetric connections is the guarantee of second feature of total input couplings. In this sense, can we extend the concept of cluster synchronization to the real brain networks? Thus, an open question is whether it is possible to form a remote synchronized cluster from the nodes with asymmetric connections but the same total input coupling.
2. **RS of star graph with the approximate same frequencies in both the hub and peripheral nodes.** In the schematic figure of Figure 8, the frequency of hub is designed to be much larger than that of peripheral nodes [73, 79]. However, in realistic networks, each individual node and its neighbors can be considered as a star network, thus both the hub and peripheral nodes should have the approximate same frequency. That is, we do not have the condition that the frequency of hub is much larger than that of peripheral nodes. Then, an open question is how to implement RS of star graphs with the approximate same frequency in both the hub and peripheral nodes.
3. **Bridging the functional and structural cognitive subnetworks.** The eigenmode analysis is currently a hot topic to reveal the physical mechanisms of cognitive brain functions. Different from the interaction of pairwise, the eigenmode analysis considers the interaction among all the network nodes. So far, a main result is that a small number of leading basic modes dominate various spontaneous activities [124, 127], thus open an avenue to study the interregional interactions in the healthy and diseased brains. However, each specific brain function is generally associated with one or a few cognitive subnetworks, which is significantly different from the whole brain network. In this sense, we need to develop the eigen‐mode analysis for the case that a few cognitive subnetworks are activated while others are inactivated. Thus, an open question is how to bridge the functional and structural cognitive subnetworks by the eigenmode analysis.
4. **Activation of cognitive subnetworks from resting state networks.** The brain is constantly receiving different signals from the outside world and thus stimulates different brain functions. For example, it was reported that the oscillatory brain activities can be stimulated by electric fields in the range of 1–2 V/m [150]. That is, each specific brain function is generally induced by specific external signals and involves only one or a few cognitive subnetworks. So far, it is not very clear how the concretive cognitive subnetworks are activated from the whole brain network and what is the influence of other cognitive subnetworks to the activation of a specific cognitive subnetwork. Toward this direction, some primary efforts have been done. For example, we designed a possible way to transmit different external signals by different detection cells [151]. Other evidences include the finding of place cell and grid cell, that is, “inner GPS” in the brain that orients us in space [152]. But they are largely insufficient to understand the activation of specific cognitive subnetworks, especially the frequently switching between them. Thus, an open question is whether we have other ways to understand the activation of cognitive subnetworks from resting state networks.
5. **Physical mechanisms of intelligence quotient (IQ).** More and more evidences have shown that IQ is closely relate not only to the size of brain and sexuality but also to the structure of the brain network. We presented a primary model to represent the trade‐off between processing efficiency and wiring cost [153]. It is revealed that the local correlations between IQ and trade‐off are significantly different from males to females, that is, males and females have different running modes [153]. However, individual’s value of IQ is very complicated and involves not only one or a few local areas but many brain regions. To understand how the structure of brain network influences its IQ is still an open question.
6. **Detection of multi‐sensing signals.** Except the weak signal detection by single signal source in Section 5, human brain usually receives multiple signals to implement one or multiple physiological functions, including perturbations or noise [154, 155]. The combination of multiple signals thus guarantees the rapid and accurate trigger of cognitive and memory functions [156]. However, current studies of signal amplification are mainly focused on single signal. In this sense, the detection and transmission of multi‐sensing signals is a promising open question.
7. **Reconstructing brain dynamic networks by a finite number of time series.** As we have pointed out in Section 1 that the neural system of human brain has a huge number of neurons [3, 4], that is, 10^11^ neurons and 10^14^ links among them. To conveniently study the dynamics of this huge number of neurons, we have to make some simplifications so that the neural network of human brain can be transformed into a structural brain network with much less size, such as the networks with 998 nodes [5, 6], 234 nodes [7], and 76 nodes [8]. Based on these structural networks, we can take time series from these nodes to construct their corresponding functional brain networks. A common point for all these brain networks with different sizes is that they are generally measured at the resting state. Then, we have an inverse problem: can we figure out the structural connections, based on the functional connections? Especially, is it possible to implement this inverse problem by using only part of the functional connections? Along this line, some primary works have been done [157, 158, 159]. The idea is to use the feature of fast‐varying noises or high‐frequency signal injection to remove the false links. How‐ever, these works only show good reconstruction at small networks. Thus, an open question is how to reconstruct brain dynamic networks by a finite number of time series?
8. **Influence of higher‐order links to the emergence of collective behaviors in brain networks.** Because of the community and rich‐club topology, the interaction of brain networks is very complicated. So far, most works on brain networks are focused on the pairwise interaction but not the higher‐order links. Recently, Mishra et al. made a primary study of eigenvectors by the higher‐order links [160]. Then, an open question is how the higher‐order links influence the dynamics of brain networks?
9. **Understanding brain functions by machine learning.** Currently, machine learning is a hot topic widely applied in different fields, although it was originally from the brain science. A main technique of machine learning is the reservoir computing (RC) [161, 162], which, driven by the input data, generates the output data through a readout function. An advantage of RC is that it is a model‐free and data‐based technique, thus can be used to the cases where prior knowledge of the network models is unavailable, including the network structure, coupling function, and oscillator dynamics. As more and more data on brain functions have been collected in experiments, machine learning may be expected to have important contributions in this field.

Except these nine main problems, there are also other problems worthy to be studied, such as the applications of current results on the modulating of cognitive performance and cross‐regional synchronization. The applications of eigenmode analysis on clinical studies are also expected in the aspects of the treatment of neurological and psychiatric disorders and diseases such as epilepsy [163], schizophrenia [164], AD [165], and depression [166].

## AUTHOR CONTRIBUTIONS

**Zonghua Liu**: Conceptualization; investigation; writing – review & editing.

## CONFLICT OF INTEREST STATEMENT

The authors declare that they have no known competing financial interests or personal relationships that could have appeared to influence the work reported in this paper.

## Data Availability

No data were used for the research described in the article.
